# Chronological expression and distribution of African swine fever virus p30 and p72 proteins in experimentally infected pigs

**DOI:** 10.1038/s41598-022-08142-y

**Published:** 2022-03-09

**Authors:** Taehwan Oh, Duy Tien Do, Danh Cong Lai, Lan Thi Nguyen, Joo Young Lee, Phan Van Le, Chanhee Chae

**Affiliations:** 1 Department of Veterinary Pathology, College of Veterinary Medicine, Seoul National University, 1 Gwanak-ro, Gwanak-gu, Seoul, 08826 Republic of Korea; 2 Faculty of Animal Sciences and Veterinary Medicine, Nong Lam University, Thu Duc district, Ho Chi Minh City, Vietnam; 3 College of Veterinary Medicine, Vietnam National University of Agriculture (VNUA), Hanoi, Vietnam; 4 ChoongAng Vaccine Laboratories, Daejeon, 34055 Republic of Korea

**Keywords:** Virology, Viral pathogenesis

## Abstract

African swine fever virus (ASFV), the causative agent of contagious hemorrhagic disease in domestic pigs and wild boars, has temporally regulated gene expression kinetics. The p30 and p72 major structural proteins are involved in viral entry each with different expression kinetics, but neither of their chronological expressions and distribution have been identified in virus-infected animals. Here, we found that both transcription and translation levels of p30 were significantly higher than those of p72 in target organs during the earlier infection-phase. Lymphocyte apoptosis/necrosis and angiectasia were observed as signs of early infection with acute African swine fever. These results show that the chronologically differential expression of ASFV structural proteins tends to be prominent in infected animals, and the p30 protein could play a role in the indication of acute lesions during early infection compared to the late-expressed p72 protein. In conclusion, we propose to consider the chronological expression dynamics of ASFV structural proteins in infected animals to understand virus pathogenesis and antigen targeting for vaccine development.

## Introduction

African swine fever (ASF) virus (ASFV), the causative agent of ASF is a large, enveloped, icosahedral, double-stranded DNA virus which belongs to the genus *Asfivirus*, family *Asfaviridae* and order *Asfuvirales*^1^. The virus has a different number of open reading frames (ORFs) depending on the isolate and encodes for more than 150 proteins, many of which are highly immunogenic^2^. The complexity of ASFV lends to difficulty in understanding its viral infection mechanisms which is one of the main contributors in the hinderance of vaccine development. ASF is a contagious, hemorrhagic disease of domestic pigs and wild boars with a high mortality rate^1^. In addition to direct transmission between domestic pigs and wild boars, ASF is transmitted following a sylvatic cycle through *Ornithodoros* genus soft ticks^3^. Since its first identification in Kenya in 1921, the disease entered into the Iberian peninsula in 1957 before it spread transcontinental and into Georgia by 2007^4,5^. The disease further spread to the Russian Federation and throughout Eastern Europe before it arrived to China in 2018^6,7^. Since then, it has continued to spread throughout most of the remaining Asian countries^8,9^.

ASFV has a unique strategy of virus gene expression, which occurs through temporal regulation during mRNA transcription. There are four classes of mRNAs; immediate-early, early, intermediate and late genes according to their distinctive accumulation kinetics^10,11^. The expression of ASFV proteins follows these transcriptional kinetics, yielding structural and nonstructural proteins chronologically^12^. Structural protein p30, which is involved in virus entry, is observed from 2 to 4 h post-infection through in vitro assays, indicating the start of early virus gene expression^13,14^. Meanwhile, p72, which is critical in the formation of the major composition of the viral capsid, is expressed in late phase of virus replication^15,16^. The expression kinetics of p30 and p72 differ significantly between the cell lines^17^.

While the expression of ASFV proteins and their roles have been vastly studied in vitro at the intracellular level^13–15^, but a correlation with animal infection has not been well established. In early immunohistochemistry experiments and in situ hybridization, ASFV antigens were detected mainly in mononuclear phagocytic cells in the early stages of infection, while other cell types such as endothelial cells, epithelial cells and hepatocytes were detected in the later stage of infection^18,19^. Expression of early protein p30 and late protein p72 is well established^13–16^ and widely used for in vitro studies of temporal viral transcription and protein synthesis^17,20^. However, studies on the differential expression patterns of p30 and p72, and the cells expressing these structural proteins have yet to be conducted according to disease course in ASFV-infected pigs. Therefore, the objective of the present study was to design a temporal pathology model of acute ASF to investigate the chronological expression and distribution of ASFV structural proteins in the progress of lesion development.

## Results

### Clinical observations

**Figure 1:**
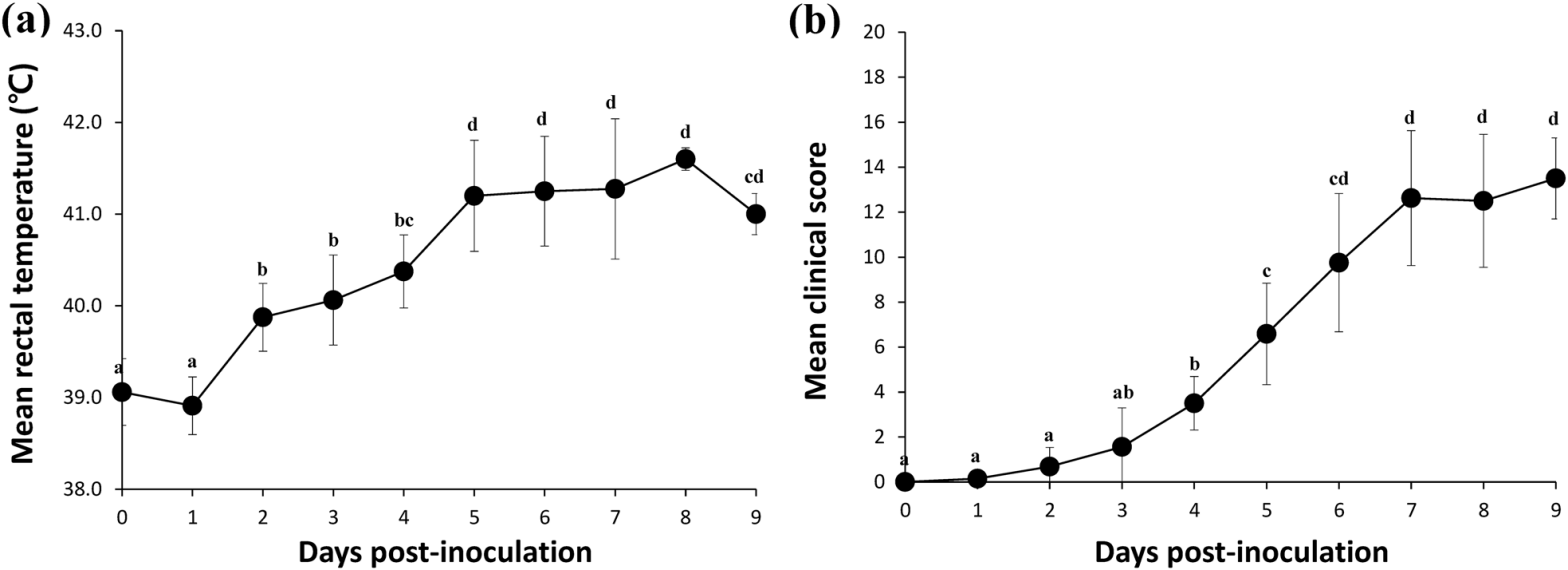
Mean rectal temperature (**a**) and mean clinical scores (**b**) of the infected pigs. Variation is expressed as the standard deviation. Different superscripts (a, b, c, and d) indicate significant (*P* < 0.05) difference between the results of different dpi.

The pigs were inoculated orally with 3 mL of highly virulent ASFV strain D/VN/BD/2019 (1 × 10^4^ TCID_50_/ml). The mean rectal temperature of ASFV-infected pigs slightly decreased between 0 to 1 dpi, and significantly increased (*P* < 0.05) at 2 dpi. At 5 dpi, the mean rectal temperature was above 41 °C, significantly increased (*P* < 0.05) from earlier dpi, at which time clinical signs were also observed. Afterward, the mean rectal temperature reached its maximum at 8 dpi (41.6 ± 0.1 °C), before decreasing at 9 dpi followed by death (Fig. 1a). The mean clinical score of ASFV-infected pigs increased gradually throughout the experiment (Fig. 1b). At 4dpi, 5dpi, and 7dpi, there was a significant (*P* < 0.05) increase in clinical score compared to the earlier dpi, respectively. Anorexia and recumbence were the first clinical signs of infection. The predominant lesions which attributed to an increase in clinical scores were joint swelling and ocular discharge followed by cyanosis. Symptoms related to respiratory (coughing) and digestive (diarrhea) findings were not clear in most of the pigs.

### Viremia and seroconversion

**Figure 2:**
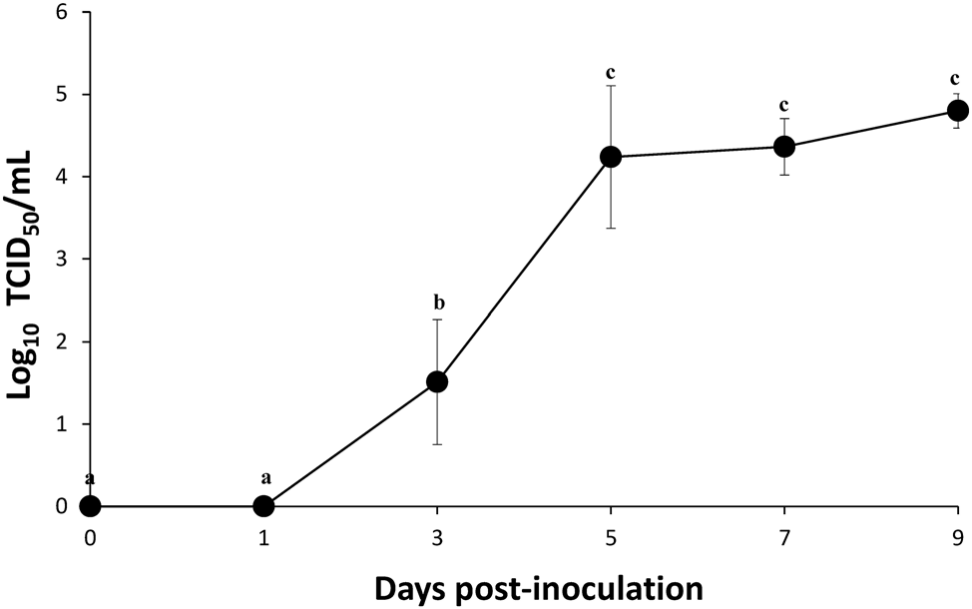
Viremia of the infected pigs. Results were shown as log_10_ TCID_50_/mL. Different superscripts (a, b, and c) indicate significant (*P* < 0.05) difference between the results of different dpi.

Viremia appeared at 3 dpi, and significantly increased (*P* < 0.05) in all pigs at 5 dpi. The mean viral load in whole blood then plateaued until the end of the experiment at 9 dpi (Fig. 2). Seroconversion was measured in the blood by commercial ELISA kit. All pigs were seronegative throughout the experiment. Only one pig at 9 dpi exhibited a borderline measurement (30% < S/P percent < 40%). Since anti-p30 antibodies can be detected by an optimized ELISA from 8–12 dpi under experimental condition^21^, it can be expected that this pig was at the onset of seroconversion.

### Quantification of cDNA copies of ASFV p30 and p72 in infected tissues

**Figure 3:**
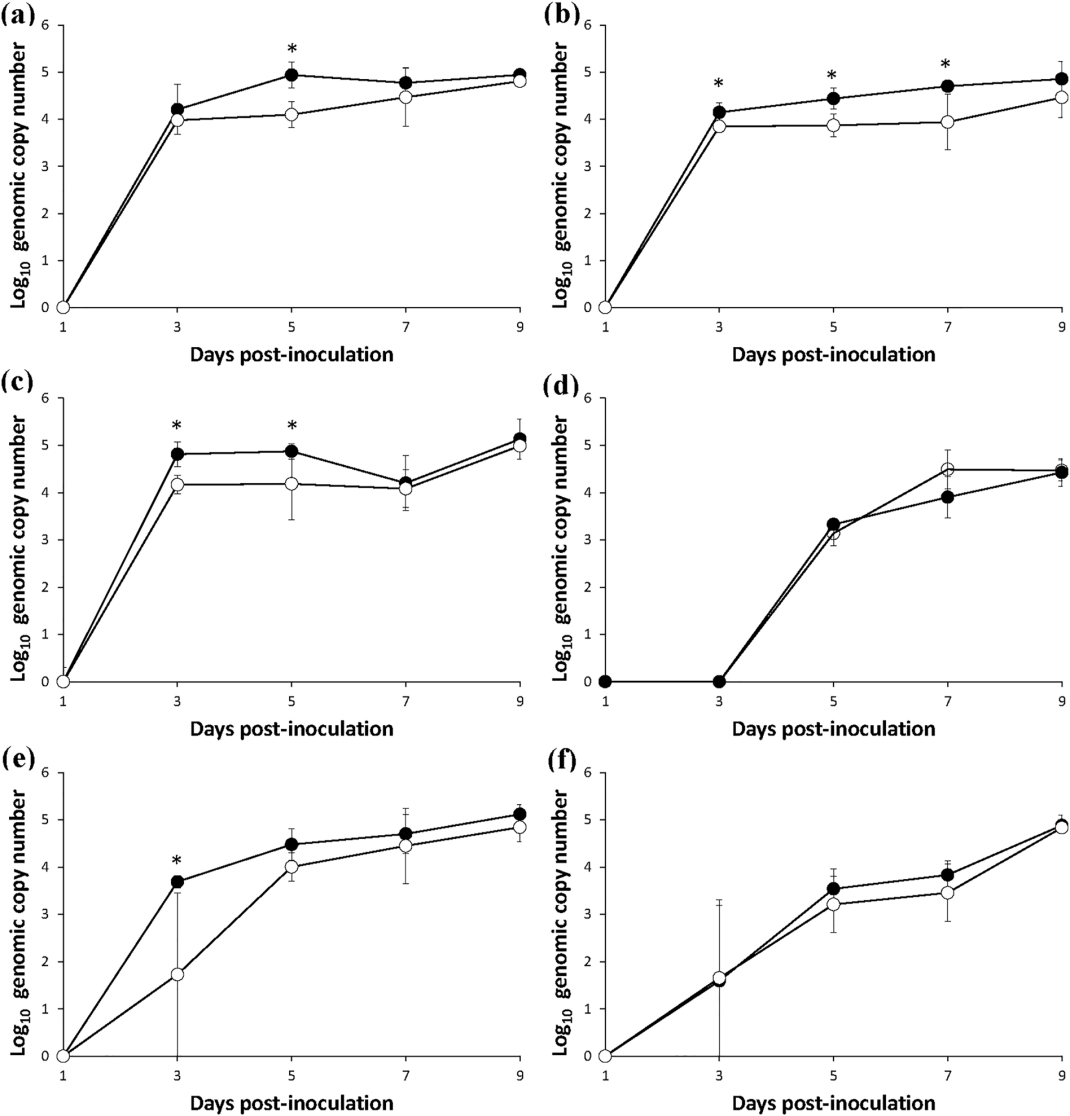
Mean values of the genomic copy number of ASFV structural protein p30 (●) and P72 (○) in the lung (**a**), liver (**b**), spleen (**c**), kidney (**d**), lymph node (**e**) and thymus (**f**) of infected pigs. Variation is expressed as the standard deviation. Marks (*) indicate significant (*P* < 0.05) difference between 2 protein genes.

Within the lungs, the p30 copy numbers were significantly higher (*P* < 0.05) than the p72 copy numbers at 5 dpi (Fig. 3a). Within the liver, the p30 copy numbers were significantly higher (*P* < 0.05) than the p72 copy numbers at 3, 5 and 7 dpi (Fig. 3b). Within the spleen, the p30 copy numbers were significantly higher (*P* < 0.05) than the p72 copy numbers at 3 and 5 dpi (Fig. 3c). There were no significant differences between p30 and p72 copy numbers within the kidney or thymus (Fig. 3d, f). Within the lymph node, the p30 copy numbers were significantly higher (*P* < 0.05) than the p72 copy numbers at 3dpi (Fig. 3e).

### Quantification of antigen positive cells of ASFV p30 and p72 in infected tissues

**Figure 4:**
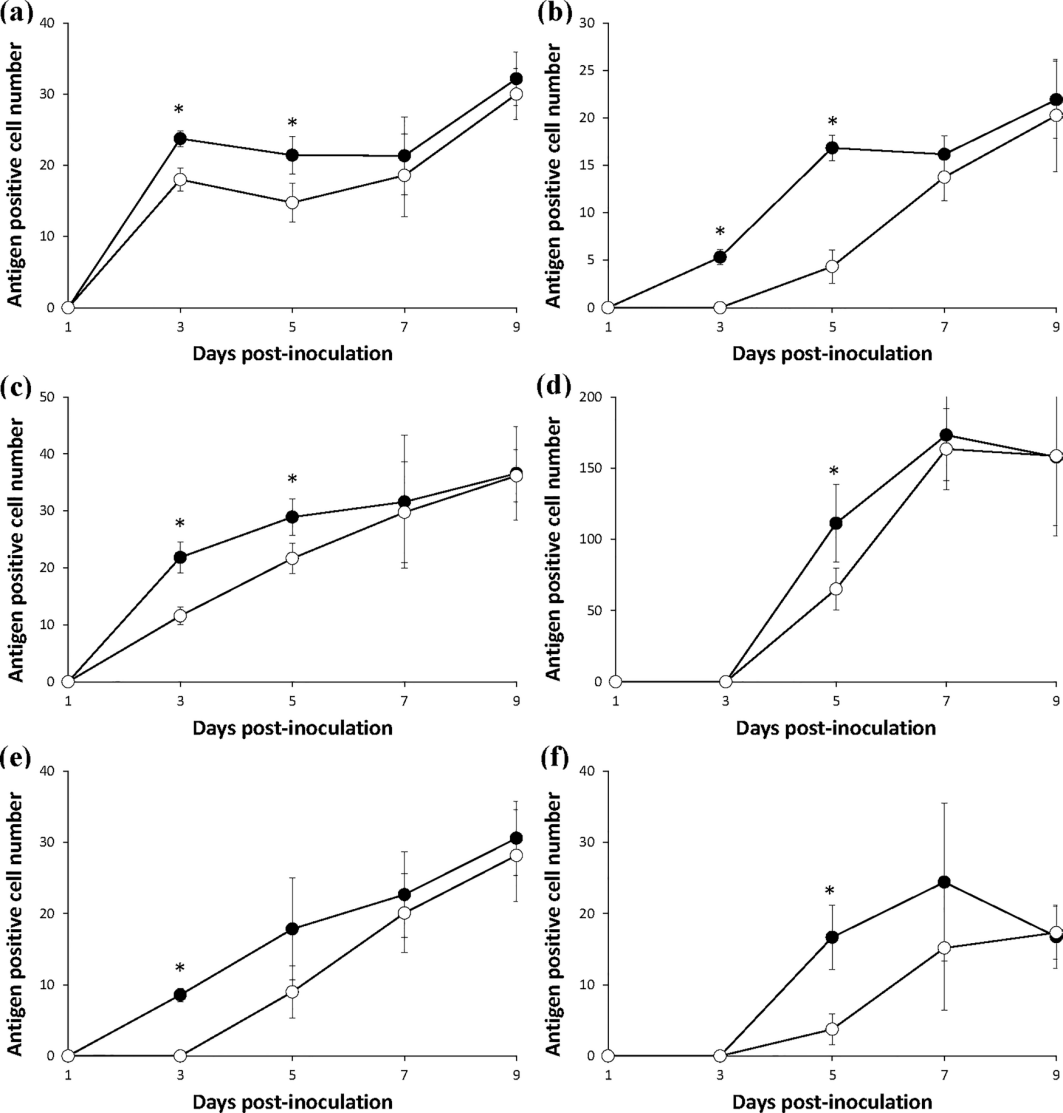
Mean values of the antigen positive cell number of ASFV structural protein p30 (●) and P72 (○) in the lung (**a**), liver (**b**), spleen (**c**), kidney (**d**), lymph node (**e**) and thymus (**f**) of infected pigs. Variation is expressed as the standard deviation. Marks (*) indicate significant (*P* < 0.05) difference between 2 proteins.

**Figure 5:**
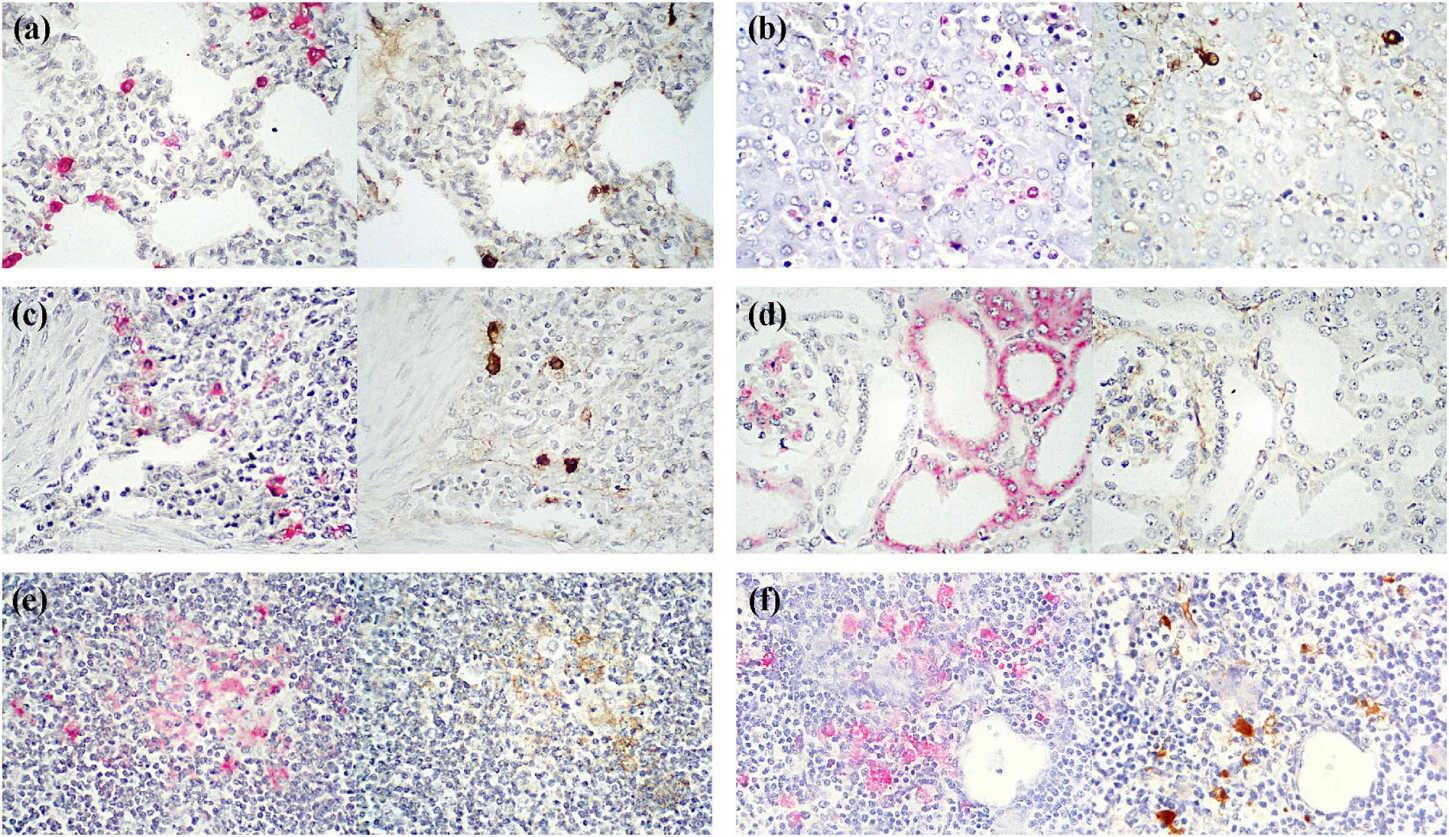
IHC on serial sections of the lung (**a**), liver (**b**), spleen (**c**), kidney (**d**), lymph node (**e**) and thymus (**f**) from 5 dpi necropsied pigs. The immunoreactive signals of p30 and p72 were seen as red and brown grains respectively.

There was significantly higher (*P* < 0.05) number of p30-positive cells than p72-positive cells in the lung, liver and spleen at 3 and 5 dpi (Fig. 4a–c and Fig. 5a–c). The kidney also contained a significantly higher (*P* < 0.05) number of p30-positive cells than p72-positive cells at 5 dpi (Figs. 4d and 5d). The lymph node followed suite with significantly higher (*P* < 0.05) number of p30-positive cells than p72-positive cells at 3 dpi (Figs. 4e and 5e). Finally the thymus contained a significantly higher (*P* < 0.05) number of p30-positive cells than p72-positive cells at 5 dpi (Figs. 4f and 5f).

### Correlation between the number of cDNA copies and antigen-positive cells in ASFV proteins in infected tissues

Significant positive correlations were revealed between p30 cDNA copies and p30 antigen-positive cells in lung (R = 0.880, *P* < 0.01), liver (R = 0.797, *P* < 0.01), spleen (R = 0.781, *P* < 0.01), kidney (R = 0.881, *P* < 0.01), lymph (R = 0.827 , *P* < 0.01) and thymus (R = 0.677 , *P* < 0.01) tissues over time. There were also positive correlations between p72 cDNA copies and p72 antigen-positive cells in lung (R = 0.815, *P* < 0.01), liver (R = 0.530 , *P* < 0.05), spleen (R = 0.809, *P* < 0.01), kidney (R = 0.915, *P* < 0.01), lymph (R = 0.773 , *P* < 0.01) and thymus (R = 0.669 , *P* < 0.01) tissues over time.

### Immunohistochemistry

**Figure 6:**
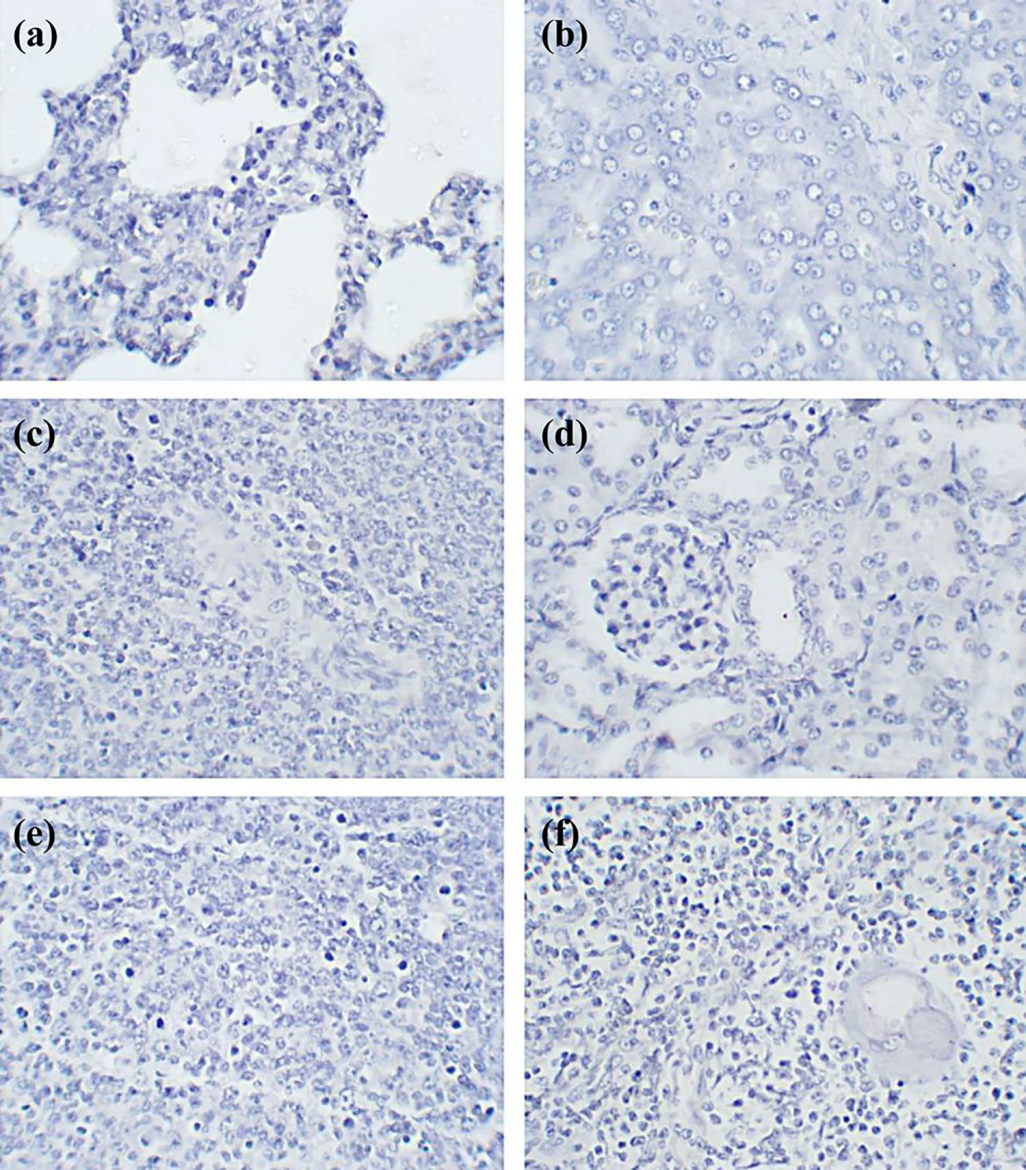
IHC on tissue sections of the lung (**a**), liver (**b**), spleen (**c**), kidney (**d**), lymph node (**e**) and thymus (**f**) from 10 weeks old growing pig served as negative controls. No immunoreactive signals were seen in all 6 tissues.

Both p30 and p72 were detected in lung pulmonary intravascular macrophages and interstitial mononuclear cells, with p72 at a lesser degree (Fig. 5a). Within the liver, circulating monocytes and kupffer cells tested positive for p30 and p72 antigens, with p72 at a lesser degree than p30 (Fig. 5b). Within the spleen, macrophages in red pulp and white pulp were immunoreactive with both p30 and p72 antigens, with p72 at a lesser degree (Fig. 5c). Within the kidney, immunoreactivity of both p30 and p72 were observed in tubular epithelial cells, also with p72 at a lesser degree (Fig. 5d). Within both lymph nodes and the thymus, macrophages tested positive for both p30 and p72, with p72 at a lesser degree than p30 (Fig. 5e, f). There were no immunoreactivity of both p30 and p72 in all 6 tissues of negative controls (Fig. 6a–f).

### Histopathology

At 5 dpi, the severity of ASFV-associated microscopic lesions observed in lung, liver, spleen, kidney and thymus were significantly increased (*P* < 0.05) from lesions observed at 1 and 3 dpi. The microscopic lymph node lesion score increased significantly (*P* < 0.05) at 5 dpi from 1 dpi. At 7 dpi, only hepatic lesion scores increased significantly from 5 dpi (*P* < 0.05). The severity of ASFV-associated microscopic lesions observed in the liver, kidney, lymph node and thymus significantly increased (*P* < 0.05) at 9 dpi from lesions observed at 5 dpi and 7 dpi. Microscopic splenic lesion score significantly increased (*P* < 0.05) at 9 dpi from 5 dpi (Table 1).

**Table 1:** Mean microscopic lesion scores of tissue samples in ASFV infected pigs at 1,3,5,7 and 9 dpi.

dpi	1	3	5	7	9
Lung	0.75 ± 0.43^a^	1.17 ± 0.17^a^	1.67 ± 0.24^b^	1.58 ± 0.14^b^	1.67 ± 0.24^b^
Liver	0.63 ± 0.41^a^	1.25 ± 0.25^a^	1.88 ± 0.22^b^	2.38 ± 0.22^c^	2.88 ± 0.22^d^
Spleen	0.81 ± 0.11^a^	0.88 ± 0.22^a^	1.75 ± 0.35^b^	2.38 ± 0.28^bc^	2.82 ± 0.32^c^
Kidney	0.69 ± 0.11^a^	0.69 ± 0.19^a^	1.25 ± 0.31^b^	1.31 ± 0.11^b^	1.81 ± 0.27^c^
Lymph node	0.67 ± 0.33^a^	1.08 ± 0.14^ab^	1.33 ± 0.24^b^	1.67 ± 0.24^b^	2.67 ± 0.24^c^
Thymus	0.50 ± 0.29^a^	0.75 ± 0.14^a^	1.17 ± 0.17^b^	1.50 ± 0.17^b^	2.17 ± 0.17^c^

*Variation is expressed as the standard deviation. Different letters a, b, c in the same line mean statistical significance.

## Discussion

In the present study, chronological expression kinetics of ASFV genes were confirmed with differential expression of the early protein p30 and the late protein p72 in experimentally infected pigs. The two structural protein expression levels were significantly different in the early stage of the infection, where acute ASF lesions develop. The differential expression of p30 and p72 was demonstrated in transcription levels measured by qRT-PCR and translation levels measured by IHC, with a significant positive correlation between the two levels. These results provide scientific evidences that ASFV is able to replicate and produce structural p30 and p72 proteins chronologically in target cells of infected animals.

Both expression of p30 and p72 showed consistent results at the transcriptional and translational levels in all infected tissues except kidney and thymus. The number of cDNA copies and antigen-positive cells of p30 were significantly (*P* < 0.05) higher than those of p72 in lung, liver, spleen and lymph node tissues during the early phase of infection (3 dpi, 5 dpi, or both). Within the kidney and thymus, antigen-positive cell numbers of p30 were significantly (*P* < 0.05) higher than those of p72 at 5 dpi, but there were no significant differences in cDNA copy numbers. It is possible that relatively lower cDNA copies of ASFV during the early infection phase of kidney and thymus tissues resulted in these different results between the tissues. In the late phase of acute infection (7 or 9 dpi), there were no significant difference in p30 or p72 expression levels in any of the infected tissues. We found that p72, which is classified as a late protein at the intracellular level, is fully expressed and distributed at later stages of the disease in infected animals compared to p30.

The temporal pathology evaluation of this study revealed that infected animals developed lymphocyte apopotosis/necrosis and angiectasia in target organs from 3 dpi. This became apparent at 5 dpi and thereafter, as observed through the increase of lesion severity. It is generally accepted that primary ASFV-associated lesions such as lymphoid destruction or tissue hemorrhages are mediated by cytokines secreted from infected mononuclear phagocytic cells^22–24^. Interestingly, the expression pattern of ASFV structural proteins p30 and p72 in mononuclear cells was markedly differed during the early phase (3–5 dpi) of infection where the lesions and clinical signs became evident. These results suggest a chronologically differential expression of the ASFV proteins may be the underlying mechanism of ASF pathogenesis. Both p30 and p72 proteins are involved in virus entry in that they play important roles in viral internalization and attachment respectively^25–27^, but they have different expression kinetics during the virus infection cycle^17,20^. We found that the differential expression of ASFV proteins through temporal regulation identified in vitro tends to be prominent in vivo as well, especially in the fulminant course of acute ASF. Therefore, it can be concluded that p30, which begins early post-infection and is continuously expressed throughout the infection cycle plays a role in the indication of ASF lesions rather than p72, which is expressed later post-infection^14^. It is further supported by previous findings that a delay of 2 to 4 days for p30 maximal expression was observed in attenuated viral infection compared with virulent viral infection^28^.

This study used the highly virulent ASFV strain causing acute ASF in virus-infected pigs. Since significant differences between p30 and p72 expression were found only in the early stage of acute ASF, different results may appear in experiments using lower virulent strains causing subacute or chronic ASF. Therefore, further experiments using various strain of ASFV should be taken into consideration to establish chronological expression and distribution of viral structural proteins in ASF pathogenesis.

Through this pathological study, the chronological expression and distribution of ASFV structural proteins were demonstrated in experimentally infected pigs. We revealed that the viral structural protein p30, which is expressed earlier compared to the major capsid protein p72, could serve as an indicator of the acute course of the disease in ASFV-infected animals. Thus, it is necessary to consider the chronological expression dynamics of ASFV structural proteins in infected animals to understand virus pathogenesis and antigen targeting for vaccine development. Further experimental studies are needed to investigate a hitherto unknown expression pattern of ASFV structural proteins in vivo.

## Methods

### Virus

ASFV field isolate D/VN/BD/2019 was the inocula strain used in this study. This strain belongs to genotype II and was isolated from the tissues of pig suffering from acute ASF in Vietnam^29,30^. A sequence of p30 (MW039155), p54 (MW039156), and p72 (MW039157) isolate genes were 100% identical to those of isolates from Georgia and China^5,7^. Virus stocks were grown in porcine alveolar macrophages (PAMs), and confirmed by immunocytochemistry with specific antibody against p30 (Alpha Diagnostic Intl. Inc., San Antonio, Texas, USA). The virus was titrated according to the Reed and Muench method by tissue culture infective dose prior to inoculation into pigs^31^.

### Animals

Twenty four clinically healthy, cross-bred, 10 weeks old growing pigs weighing 20 kg were purchased from a commercial swine farm. Pigs were tested and confirmed negative for ASFV by antibody ELISA and antigen PCR as recommended by the Office International des Epizooties (OIE, Paris, France). Study pigs were also negative for additional pathogens such as foot-and-mouth disease virus, classical swine fever virus and porcine reproductive and respiratory syndrome virus^32–34^.

### Experimental design

This study evaluated twenty experimentally infected pigs along with four naïve sentinel pigs. All non-sentinel pigs were inoculated orally with 3 mL of ASFV (1 × 10^4^ TCID_50_/ml), while the sentinels were similarly inoculated with an equal volume of PBS. Upon challenge, the pigs were randomly assigned into 4 pens, each containing six animals. One sentinel pig was assigned to each of the four pens. Following ASFV challenge, the physical condition and the rectal temperature of each pig were monitored daily. Blood samples from all pigs were collected by jugular venipuncture at 0, 1, 3, 5, 7, 9, 14, 16 and 18 days post-inoculation (dpi). Four pigs were sacrificed at 1, 3, 5 and 7 dpi respectively. An additional four pigs which had been scheduled for sacrifice at 10 dpi were found dead at 9 dpi. Two moribund sentinel pigs were euthanized at 16 dpi following endpoint criteria previously described^35^ and the remaining two sentinels were found dead at 18 dpi. Lung, lymph node, heart, liver, kidney, urinary bladder, spleen, thymus, brain, bone marrow, stomach and intestine from each pig were collected for further analysis. The tissues were individually collected under clean conditions, stored and tested separately.

### Clinical observations

The pigs were monitored daily for rectal temperature and clinical symptoms by the same personnel. Clinical scores were estimated by summing the score of seven clinical signs (anorexia, recumbence, skin hemorrhage/cyanosis, joint swelling, labored breathing or coughing, ocular discharge and digestive findings) as previously described^36^. Scoring was done about follows : 0 = absent, 1 = mild; 2 = moderate; 3 = severe.

### Viremia and seroconversion

All blood samples collected throughout the study were tested for presence of infectious ASFV, and the viremia of infected pigs were expressed as log_10_ TCID_50_/mL determined by titration on PAMs^20^. The serum samples were also tested for specific ASFV antibodies using a commercially available ASFV ELISA (ID Screen® African Swine Fever Indirect, IDvet, Grabels, France) which detects antibodies against the viral proteins p32, p62 and p72. All serum samples were tested in duplicate.

### Quantitative reverse transcription polymerase chain reaction

Quantitative reverse transcription polymerase chain reaction (qRT-PCR) for p30 and p72 transcription were performed as previously described with slight modification^17,20^. Briefly, total RNA was extracted from collected tissue homogenates using RNeasy Mini Kit (Qiagen, Hilden, Germany) and RNase-Free DNase Set (Qiagen). Reverse transcription was done with SuperScript™ III First-Strand Synthesis SuperMix (Invitrogen, Carlsbad, CA, USA) according to the manufacturer’s instructions. The Real-time PCR was carried out using a Mic Real-Time PCR Detection System (Bio Molecular Systems, Upper Coomera, Queensland, Australia) where the conditions were 95 C for 5 min, followed by 40 cycles of 95 C for 15 s and 60 C for 40 s. Quantification of cDNA copy number of each viral genes were achieved by standard curves constructed by ten-fold serial dilutions of the standard templates. Only data from qRT-PCR showing an amplification efficiency of ≥ 0.95 and an R^2^ value of ≥ 0.98 were used in the analysis. The copy number of ASFV p30 and p72 in the tissues were expressed as log_10_ value. Each cDNA was quantified by three independent qRT-PCR to further determine their correlation with antigen-positive cell numbers.

### Immunohistochemistry

Immunohistochemistry (IHC) for the analysis of ASF viral protein p30 and p72 expression in serial sections of infected tissues was performed using commercially available specific antibodies as previously described with slight modification^37,38^. The rabbit polyclonal ASFV phosphoprotein p30 antibody (Alpha Diagnostic Intl. Inc., San Antonio, Texas, USA), and the mouse monoclonal ASFV phosphoprotein p72 antibody (Ingenasa, Madrid, Spain) were used for IHC. Differential colorization steps were applied for visualization of two antigens, which consisted of alkaline phosphatse (AP) staining for p30 and diaminobenzidine (DAB) staining for p72 (Dako, Glostrup, Denmark). The expression of each viral proteins were analyzed morphometrically as previously described^37,39^. To obtain quantitative antigen amount from each slide of tissues, ten fields were randomly selected, and the number of positive cells per unit area (0.25mm^2^) was counted. Mean values were then calculated. Lung, liver, spleen, kidney, lymph node and thymus tissue specimens from a 10 weeks old growing pig negative with ASFV ELISA and PCR served as negative tissue controls. The tissue specimens from a 7 dpi pig equally expressing p30 and p72 antigens served as positive tissue controls.

### Histopathology

Morphometric analysis of histologic lesions was performed blindly by two pathologists (T. Oh and C. Chae) following standardized guidelines of experimental ASFV infections with slight modifications^30,35^. Microscopic evaluation was performed on lung, liver, spleen, kidney, thymus, and lymph nodes (inguinal, gastrohepatic, renal). The scores were given as 0 = absent, 1 = mild, 2 = moderate, and 3 = severe for each organ. Microscopic pulmonary lesions were scored for alveolar and interstitial edema, peribronchial hemorrhages and inflammatory cell infiltration. Hepatic lesions were scored for hepatocellular degeneration, portal angiectasia with sinusoidal dilation, and peribiliar edema with hemorrhage. Splenic lesions were scored for lymphoid and histiocytic necrosis, angiectasia and congestion. Renal lesions were scored for cortex and medullar hemorrhage, tubular necrosis with hyaline cast, and interstitial mononuclear inflammation. Lymph nodes were scored for lymphoid depletion, histiocytic necrosis and hemorrhages. Thymic lesions were scored for lymphoid depletion and histiocytosis.

### Statistical analysis

Mann–Whitney test was used to examine whether there were statistically significant differences between p30 and p72 in the number of genomic copies and antigen-positive cells from ASFV infected tissues. Pearson's correlation coefficient was calculated to assess the relationship between the number of genomic copies and antigen positive cells. A value of *P* < 0.05 indicated a statistical significance. The Kruskal–Wallis test was performed on data measured by clinical observations and histopathology at different dpi. The Kruskal Wallis test results which showed statistical significance were further evaluated by Mann–Whitney test to compare the differences in the results. Results were reported in *P*-value where a value of *P* < 0.05 was considered to be significant.

### Ethical approval

This experimental study followed the guidelines approved by the Animal Ethics Committee of the Vietnam National University of Agriculture (Approval number, 5112/QĐ-NNH; approval date, December 31, 2019). And all authors complied with the ARRIVE guidelines.

## Data Availability

The datasets used and/or analyzed during the current study are available from the corresponding author on reasonable request.
